# Gallic acid alleviates visceral hyperalgesia following maternal separation in mice by inhibiting EphrinB2/EphB2 signaling mediated activation of neurons and glial cells

**DOI:** 10.3389/fimmu.2025.1698744

**Published:** 2025-11-27

**Authors:** Shu-fen Guo, Yu Wang, Han Zheng, Hong-qin Tu, Yu-qing Xi, Dan Li, Zheng-liang Ma, Wei Zhang, Jia-ping Ruan

**Affiliations:** 1 Department of Anesthesiology, Nanjing Drum Tower Hospital Clinical College of Nanjing University of Chinese Medicine, Nanjing, Jiangsu, China; 2 Department of Anesthesiology, Nanjing Drum Tower Hospital, Affiliated Hospital of Medical School, Nanjing University, Nanjing, Jiangsu, China; 3 Department of Anesthesiology, Nanjing Drum Tower Hospital, Affiliated Hospital of Medical School, Nanjing Medical University, Nanjing, Jiangsu, China

**Keywords:** visceral hypersensitivity, early life stress, maternal separation, ephrinB2/ephB2, NMDA, gallic acid

## Abstract

**Background:**

Early life stress (ELS) causes functional gastrointestinal issues linked to visceral hyperalgesia. Activation of spinal neurons and glial cells is key to the development and persistence of visceral hyperalgesia. Our previous research has shown that EphrinB2/EphB2 signaling in the spinal cord facilitates this hyperalgesia through neuron and glial cell activation. Gallic acid (GA), a natural compound with recognized anti-inflammatory and analgesic effects, may attenuate visceral hyperalgesia. This study investigates whether GA mitigates visceral hyperalgesia induced by ELS in mice via inhibiting EphrinB2/EphB2-mediated activation of neurons and glial cells.

**Methods:**

We employed a maternal separation (MS)-induced ELS model and recorded abdominal withdrawal reflex (AWR) scores following colorectal distension (CRD) in adult mice. Molecular docking analysis was used to evaluate the binding stability of GA with the EphrinB2-EphB2 complex or EphrinB2 alone. After CRD, we assessed EphrinB2 and EphB2 expression, glial and neuronal activation, and synaptic plasticity in the spinal cord of MS mice, with or without GA treatment. C-fos levels were measured via immunohistochemistry, and protein expression was quantified by Western blotting. EphrinB2/EphB2 co-expression with neurons or glial cells was examined by double-labeling, and a 3D reconstruction confirmed cell type-specific expression.

**Results:**

Molecular docking confirmed that GA binds stably to EphrinB2-EphB2 complex or EphrinB2 alone. In adult MS mice, CRD stimulation induced pain behaviors, accompanied by substantial activation of spinal neurons and glial cells, as well as upregulation of synaptic N-methyl-D-aspartate receptors (NMDARs). EphrinB2 and EphB2 were localized within spinal astrocytes, microglia, and neurons. Furthermore, exogenous EphrinB2 induced the activation of glial cells and neurons, NMDARs phosphorylation, and visceral hypersensitivity in naive mice. Intraperitoneal injection of GA can alleviate the above conditions.

**Conclusions:**

Our findings suggest that spinal EphrinB2/EphB2 signaling is crucial in the development of maternal separation-induced visceral hyperalgesia. GA may alleviate hyperalgesia by inhibiting the EphrinB2/EphB2 signaling pathway, thereby modulating nociceptive processing by MS.

## Hypothesis Illustration

**Figure:**
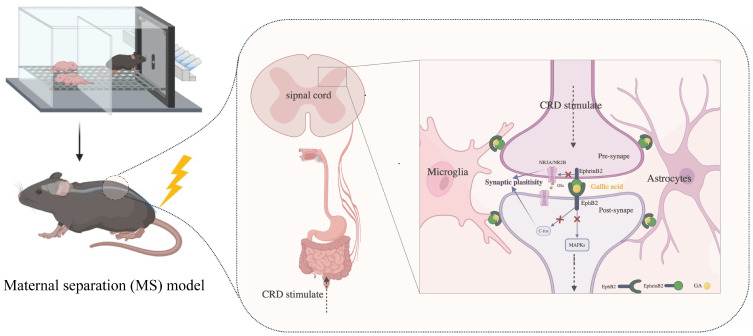
Schematic diagram illustrating the hypothesis of "Gallic acid alleviates visceral hyperalgesia in maternally separated mice by reducing the activation of neurons and glial cells through the inhibition of EphrinB2/EphB2 signaling in the spinal cord. Created with BioRender.com.

## 1 Introduction

The symptoms of irritable bowel syndrome (IBS), a functional gastrointestinal illness, include pain, distension in the abdomen, and changes in bowel habits (1). Epidemiological studies estimate that IBS affects approximately 3.8% to 4.8% of the global population (2). A key feature of the disorder is visceral hypersensitivity, defined by an exaggerated response to colorectal distension or a reduced pain threshold, often linked to heightened neuronal excitability (3). While the exact etiology remains unclear, evidence highlights the role of psychosocial factors (3, 4), particularly early life stress, such as experiences of abuse and neglect during childhood and stress encountered in adulthood, as critical contributors to its pathophysiology.

EphrinB/EphB receptor signaling is a crucial mechanism in somatic and neuropathic pain (5, 6). Analysis of EphB receptor subtypes indicates that EphB2 expression is increased in the basolateral amygdala (BLA) during stress-induced visceral hypersensitivity (7). Activation of the EphrinB2/EphB2 pathway enhances synaptic strength and plasticity in the colon, contributing to the long-term visceral hypersensitivity observed in IBS (8). Our previous research has demonstrated that maternal separation stress during early development induces visceral hyperalgesia by increasing the expression of EphrinB2/EphB2 in the spinal cord, which in turn activates mitogen-activated protein kinase (MAPK) signaling pathways like extracellular signal-regulated kinase (ERK), c-Jun N-terminal kinase (JNK), and p38 MAP kinase (p38) (9). EphrinB2/EphB2 is expressed in astrocytes grouped in the marginal layer (10, 11) and is found in sensory neurons in the superficial layers of the spinal cord, especially medium- and small-sized neurons (9, 12). It has been well-established that Ephrin ligands are localized to presynaptic sites, whereas their corresponding receptors are primarily found at postsynaptic sites, where they play a pivotal role in the regulation of synaptic function (8, 12). Activation of the EphrinB2/EphB2 signaling pathway enhances N-methyl-D-aspartate receptors (NMDARs)-dependent synaptic function, thereby increasing stress susceptibility (7).

Gallic acid (GA, 3,4,5-trihydroxybenzoic acid) is a naturally occurring secondary metabolite present in various plants, vegetables, nuts, and fruits (11, 13). It melts in ether, glycerol, alcohol, and water (14). According to earlier research, GA offers an assortment of pharmacological qualities, such as analgesic, antibacterial, anti-inflammatory, and antioxidant actions, which may help with gut health and neuropathic pain relief (14–16). By blocking the NF-κB/MAPK signaling pathway and lowering the release of inflammatory cytokines, GA has been demonstrated in other studies to lessen pain (11, 17). Interestingly, MAPKs (ERK, JNK, p38) are predominantly expressed in EphrinB2/EphB2-positive cells within the spinal dorsal horn (9). MAPK pathways act as downstream effectors of EphB receptors, and EphB activation can be modulated via feedback control by the MAPK pathway (18). Therefore, we speculate whether GA alleviates pain by blocking the spinal dorsal horn’s EphrinB2/EphB2 signaling pathway.

To explore the effect of GA on Early life stress (ELS)-mediated visceral hyperalgesia, we constructed a maternal separation (MS) model to simulate ELS in our study. Molecular docking analysis confirmed the stable binding of GA and EphrinB2/EphB2 in their natural state. Our findings demonstrate that ELS induced visceral hyperalgesia, while GA inhibited this effect. Mechanistically, our results highlight that GA ultimately alleviates pain by modulating the EphrinB2/EphB2 signaling pathway.

## 2 Experimental method

### 2.1 Molecular docking

The structures of both the EphrinB2-EphB2 complex (PDB ID: 1KGY) and the EphrinB2 complex (PDB ID: 1KGY) were obtained from the Protein Data Bank (https://www.rcsb.org/). Furthermore, the PubChem database (https://pubchem.ncbi.nlm.nih.gov) provided the structure of GA. Docking simulations between the EphrinB2-EphB2 complex or EphrinB2 and GA were performed using the AutoDock Tool (version 4.0) software, developed by the Center for Computational Structural Biology. PyMOL (version 3.2.3) was employed to visualize the resulting conformations. Convert visualization results into 2D images using Discovery Studio.

### 2.2 Animal

A 12-hour light/dark cycle, a room temperature of 21°C ± 2°C, and unrestricted access to food and water were all part of the controlled laboratory settings used to keep pregnant C57BL/6J mice (bought from Vital River, China). With the exception of the control group, all mice underwent maternal separation for 3 hours daily from post-natal day (PND) 2 to day 15 (birth designated as day 0; separation period from 09:00 to 12:00 a.m.). On day 21, the mice were weaned and permanently separated from their mothers.

**Figure 1:**
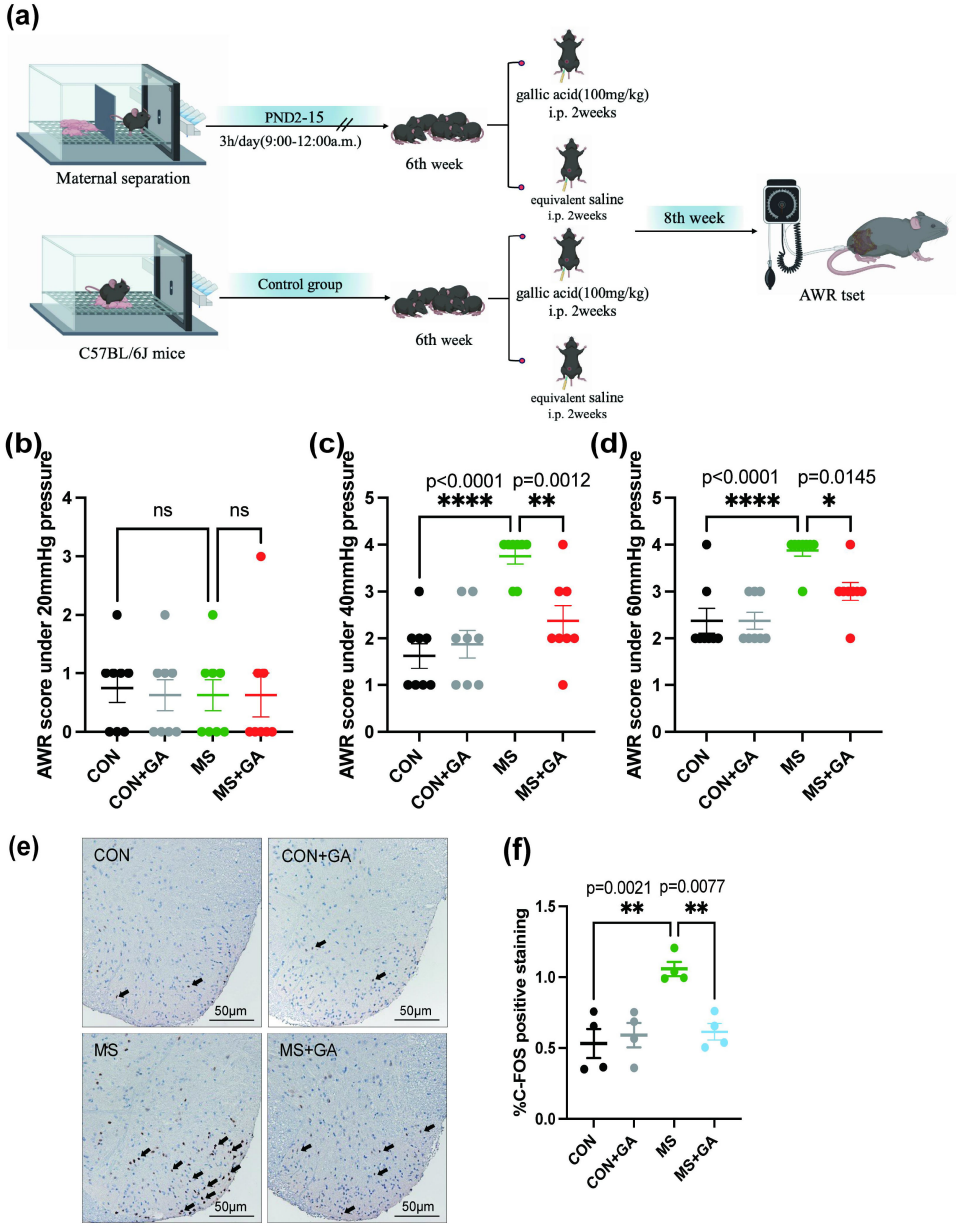
GA alleviates pain behavior in MS mice. **(a)** Timeline of MS modeling and GA treatment. **(b-d)** AWR scores measured at stimulation pressures of 20 **(b)**, 40 **(c)**, and 60 **(d)** mmHg in the control, control+GA, MS, and MS+GA groups. Data are presented as mean ± SEM (n=8 mice/group). Statistical significance was determined using a one-way ANOVA followed by Bonferroni’s *post hoc* test. *, *p* < 0.05; **, *p* < 0.01; ****, *p* < 0.0001; ns: no significance. CON+GA vs. MS: *p* < 0.05. **(e)** Representative immunohistochemistry images of the spinal dorsal horn showing positive staining for relevant markers, where the black arrows indicate c-fos. Scale bar: 50 μm. **(f)** Quantitative analysis of positive staining in spinal dorsal horn sections. Data are expressed as mean ± SEM (n=4 mice/group). *, *p* < 0.05; **, *p* < 0.01. CON+GA vs. MS: *p* < 0.05. Based on one-way ANOVA and Bonferroni multiple comparisons test. MS: Maternal separation; GA: Gallic acid; AWR: Abdominal withdrawal reflex.

This study utilized male mice for all subsequent experiments. To examine the influence of GA on visceral hyperalgesia, 46 male mice were randomized to four experimental groups: the control (CON) group, the CON group with gallic acid treatment (CON+GA), the MS group, and the MS group with gallic acid treatment (MS+GA). A saline solution was used to dissolve GA (Shanghai Macklin Biological Co., Shanghai, China). Mice in the CON+GA and MS+GA groups received intraperitoneal injections of GA at a dose of 100 mg/kg for two weeks beginning at 6 weeks of age. Meanwhile, the mice in the CON and MS groups received the same amount of saline for the same amount of time (**Figure 1a**).

### 2.3 Adult CRD

The abdominal withdrawal reflex (AWR) is used to measure how sensitive visceral organs are to colorectal distension (CRD). In brief, adult male mice are first anesthetized with isoflurane, after which a balloon catheter, pre-coated with lubricating oil, is gently inserted into the rectum and descending colon through the anus. Mice were allowed a 30-minute acclimatization period prior to testing. This ensured that all animals were fully awake, ambulatory, and displayed no signs of sedation, thereby eliminating any potential confounding influence of isoflurane on the AWR scores. The mice are positioned on a platform, and the balloon pressure is gradually increased to 20, 40, and 60 mmHg using a blood pressure monitor. The following scoring system is used to blindly evaluate the mice’s responses to graded CRD: no response to CRD (0); immobility following brief head movement (1); abdominal muscle contraction (2); abdominal lift (3); body arch and pelvic lift (4). The visceral pain threshold is subsequently determined based on these responses.

### 2.4 Tissue extraction

Mice are deeply anesthetized with isoflurane, and a sequential cardiac infusion of physiological saline followed by 4% paraformaldehyde is administered to perfuse and fix the tissues. The entire lumbosacral region is subsequently excised with care and fixed in 4% paraformaldehyde for a duration of 24 hours. Following fixation, the samples are dehydrated at 4 °C using a gradient of 15% and 30% sucrose solutions.

### 2.5 Immunohistochemistry

After being deparaffinized with xylene, tissue sections (5 μm) were rehydrated using a succession of alcohol solutions with progressively lower concentrations. The antigen repair procedure was performed at 125°C for 2 minutes, after which the tissue sections were cleaned with phosphate - buffered saline (PBS) buffers. To inhibit endogenous peroxidase activity, the sections were placed in a humidifier and treated with 3% H2O2 solution for 10 minutes at 37°C. The sections were treated for two hours with the primary antibody (C-fos, 1:300, catalog no. Ag24340, proteintech, China), then incubate with anti-rabbit Alexa Fluor 568 (1:500, catalog no. #A-11011, Invitrogen, California, USA) secondary antibodies (Invitrogen-Thermo Fisher Scientific, Milan, Italy). Reagents 1 and 2 (PV-9001, ZSGB-BIO) were then added, and each was incubated for 20 minutes at room temperature. The staining was visualized using Diaminobenzidine, after which the samples were counterstained with hematoxylin for about 1 minute, rinsed with distilled water, and finally mounted with neutral resin. A microscope from Olympus (Olympus, Japan) was used to take pictures.

### 2.6 Western-blot

The tissue samples were homogenized with protease and phosphatase inhibitors (Beyotime, P1051, China) prior to Western blot analysis. Following homogenization, the lysates were centrifuged to remove cellular debris. The Pierce BCA Protein Assay Kit (Beyotime, P0011, China) was then used to measure the amount of protein in the cleared supernatant. Protein samples were prepared for electrophoresis and stored at −80 °C until use. For SDS-PAGE, 20 µg of protein from each sample was loaded and subsequently transferred onto PVDF membranes (0.45 µm) using a transfer apparatus at 180 mA for 90 minutes. To prevent non-specific binding sites, the membranes were treated with 5% fat-free milk for one hour. They were then incubated with primary antibodies, such as EphrinB2 (1:500; Abcam, ab131536, UK), EphB2 (1:500; MABN726, Merck & Millipore, USA), GFAP (1:1000; CST, 89788T, USA), NeuN (1:1000; CST, 2430TT, USA), IBA1 (1:1000; Abcam, AB178846, UK), NR2A (1:500; Affinity, DF7955, USA), NR2B (1:1000; Abcam, ab254356, UK), phospho-NR2A (1:500; Invitrogen, PA5-105627, USA), phospho-NR2B (1:1000; Abcam, ab3856, USA), GAPDH (1:10000; Affinity, T0004, USA), and Actin (1:2000; Santa Cruz, sc-8432, USA). The membranes were rinsed three times with TBS-T the next day, with 10 minutes between washes. An HRP-conjugated or (H + L) fluorescent secondary antibody (Thermo Fisher Scientific) was then added to the membranes and incubated for 1 hour. Protein bands were found using the Tanon-4600 Chemiluminescent Imaging System (Tanon, China) after the last cycle of TBS-T washes. The results were then analyzed using ImageJ software.

### 2.7 Double-label immunofluorescence

A Leica CM3050 S Research cryostat (Leica Microsystems, Wetzlar, Germany) was used to prepare 20 μm spinal cord sections from the L6-S1 region. These sections were then maintained at -20°C until they were needed for immunofluorescence labeling. The tissue sections underwent 3 rinses with PBS to ensure thorough cleaning. After that, they were incubated for two hours at room temperature in a blocking solution that contained 2% bovine serum albumin and 0.1% Triton X-100. Following the blocking manipulate, the sections were immersed overnight at 4°C with the primary antibodies: EphrinB2 (1:250, HPA008999, Merck & Millipore, USA), EphB2 (1:250; MABN726, Merck & Millipore, USA), GFAP (1:1000, CST, 89788T, USA), NeuN (1:1000, CST, 2430TT, USA), and IBA1 (1:1000, Abcam, AB178846, UK). The next day, sections were washed three times with PBS (10 minutes each) and incubated with anti-rabbit Alexa Fluor 488 (1:1000, catalog no.#A32731, Invitrogen, California, USA) or anti-mouse Alexa Fluor 647 (1:1000, catalog no.#A32728, Invitrogen, California, USA) antibodies. Following three further PBS washes, slices were stained for five minutes using 4′,6-diamidino-2-phenylindole (DAPI). After that, an anti-fluorescence attenuation compound was used to seal the slides.

For the analysis of the spinal cord, images were captured at 20x magnification using a Leica DM6 B microscope fitted with a DFC9000 GT camera. The imaging process was conducted using the THUNDER Workstation 3D DCV, coupled with LAS X software (Leica Biosystems, Milan, Italy). Cell quantification for markers such as NeuN, GFAP, IBA1, EphB2, and EphrinB2 was carried out using ImageJ software for precise analysis. Channels were separated, regions of interest were selected, thresholds were adjusted, followed by the quantification of double-positive cells using the cell counter plugin in ImageJ. Consistent algorithms and thresholds were applied throughout the analysis process. 3D reconstructions were generated using Imaris software (version 9.1.0).

### 2.8 Statistical analysis

All experimental results are presented as the mean ± standard error of the mean. Data analysis was carried out using GraphPad Prism 9.0 (version 9.1.0). Multigroup comparisons were made using one-way analysis of variance (ANOVA) followed by a *post-hoc* test (Bonferroni test). *P* < 0.05 considered indicative of a statistically significant difference.

## 3 Results

### 3.1 Gallic acid treatment relieves pain hypersensitivity of mice

The AWR scores (**Figure 1b**) showed no statistical significance at pressures below 20 mmHg (p > 0.05). However, at pressures of 40 and 60 mmHg, the MS group’s AWR scores were significantly greater than those of the control group (p < 0.0001), indicating that the visceral hyperalgesia model had been successfully established (**Figures 1c**, **d**). After two weeks of daily intraperitoneal administration of GA, AWR scores at 40 and 60 mmHg were significantly notably lower to the MS without GA group (p < 0.05), suggesting that GA attenuated pain sensitivity in MS mice.

Since its expression is correlated with both neuronal activity and postsynaptic cytoskeletal rearrangement, C-fos, an immediate early gene, is well known as a marker of synaptic remodeling (19, 20). In response to peripheral painful stimuli, mice’s spinal dorsal horn neurons exhibit increased expression of C-fos (19). The experimental findings showed that C-fos expression was much lower after GA treatment (**Figure 1f**) and significantly greater in MS mice than in the control group (**Figure 1e**). These findings showed that GA has an impact on activity of spinal cord neurons in MS mice.

### 3.2 Effect of gallic acid treatment on EphrinB2/EphB2 receptors expression and activity

**Figure 2:**
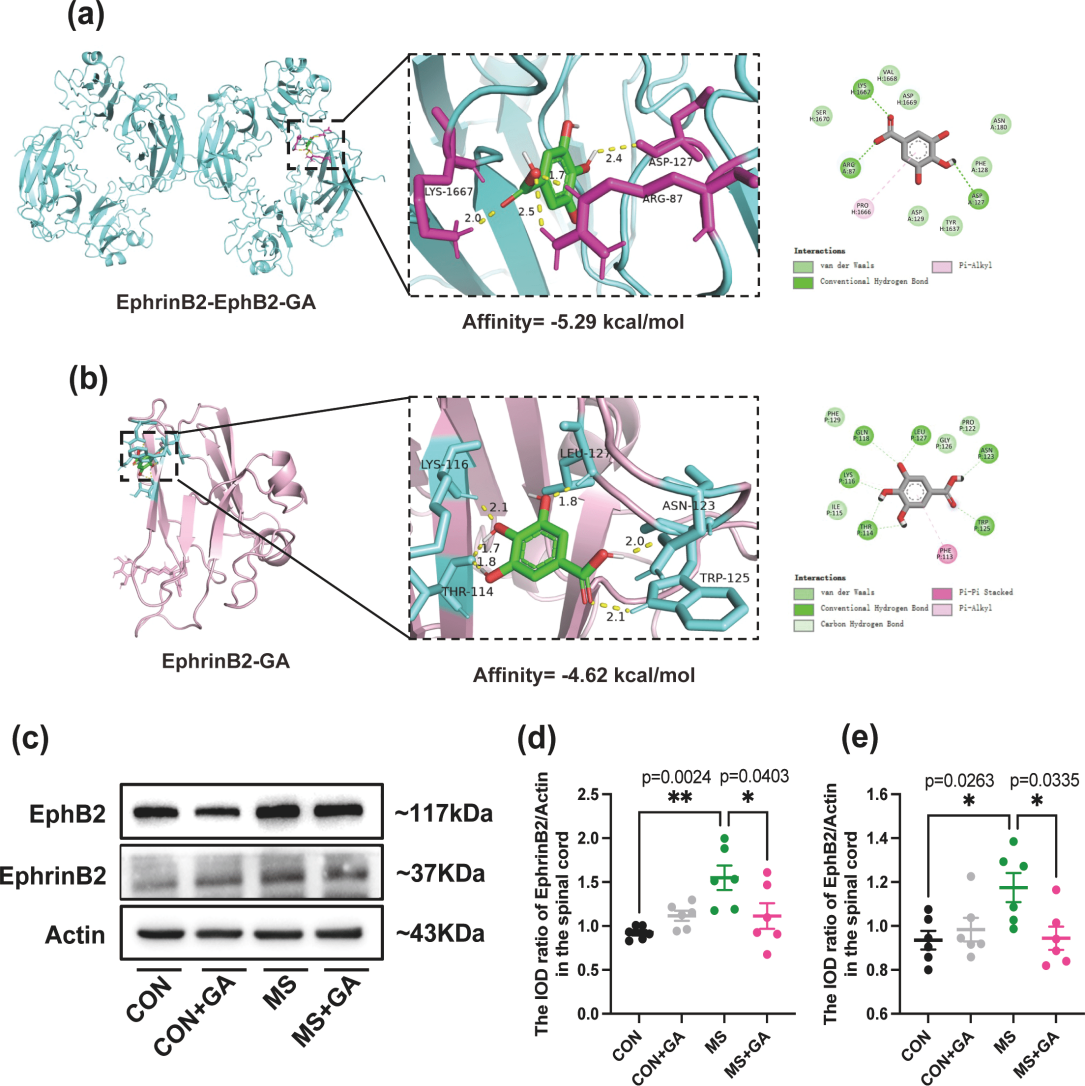
GA treatment inhibits EphrinB2/EphB2 activity. **(a)** Docking results of GA with the EphrinB2-EphB2 complex. The figure shows the 3D model structure of the EphrinB2-EphB2 complex docked with GA (left), along with a magnified view of the binding site (inset) and a 2D planar view (right). The EphrinB2-EphB2 complex is depicted in cyan, with interaction residues highlighted in red. **(b)** Docking results of GA with EphrinB2 alone. The modeled 3D structure of EphrinB2 docked with GA (left), along with a magnified view of the binding site (inset) and a 2D planar view are shown (right). GA is represented in green, EphrinB2 in pink, and interaction residues in cyan. Bond interactions are illustrated with yellow dotted lines, and bond lengths are annotated. **(c-e)** Western blot analysis and quantification of relative gray values for EphrinB2 and EphB2 levels in the spinal cords of control, control+GA, MS, and MS+GA groups. Representative blots **(c)** and quantitative analysis of EphrinB2 **(d)** and EphB2 **(e)** levels are shown. Data are presented as mean ± SEM (n=6 mice/group). Statistical significance was determined by one-way ANOVA with Bonferroni’s *post hoc* test (multiple groups). *, *p* < 0.05; **, *p* < 0.01. CON+GA vs. MS: *p* < 0.05. GA: Gallic acid.

Molecular docking were conducted to investigate the binding affinity of GA with the EphrinB2-EphB2 complex (**Figure 2a**). The docking results indicate an optimal binding affinity of 5.29 kcal/mol for GA with the EphrinB2-EphB2 complex. GA interacts with the complex by forming four hydrogen bonds with amino acid residues LYS-1667, ARG-87, and ASP-127, as well as van der Waals and Pi-Alkyl interactions with other relevant residues. Specifically, the bond distance between LYS-1667 and GA is 2.0 Å; the bond distances to ARG-87 are 1.7 Å and 2.5 Å, and the bond distance to ASP-127 is 2.4 Å (**Figure 2a**).

In parallel, molecular docking of GA with EphrinB2 alone was performed (**Figure 2b**). The results show a binding affinity of 4.62 kcal/mol for GA with EphrinB2. GA forms six hydrogen bonds with amino acid residues LYS-116, LEU-127, ASN-123, TRP-125, and THR-114, with bond distances as follows: LYS-116 at 2.1 Å, LEU-127 at 1.8 Å, ASN-123 at 2.0 Å, TRP-125 at 2.1 Å, and THR-114 at 1.7 Å and 1.8 Å (**Figure 2b**). Additionally, GA interacts with other relevant residues through van der Waals forces, Pi-Pi stacking, carbon-hydrogen bonds, and Pi-Alkyl interactions.

Consistent with these docking results, an upregulation of EphrinB2 and EphB2 was observed in MS mice, whereas this expression was significantly downregulated in GA-treated MS mice (p < 0.05) (**Figures 2c-e**), with Actin as a loading control. These findings suggest that GA may directly modulate EphrinB2/EphB2 signaling pathway activity.

### 3.3Three-dimensional reconstruction of EphrinB2/EphB2 and spinal cord neurons or glial cells, and the effect of GA on the expression of glial cells and neurons

**Figure 3:**
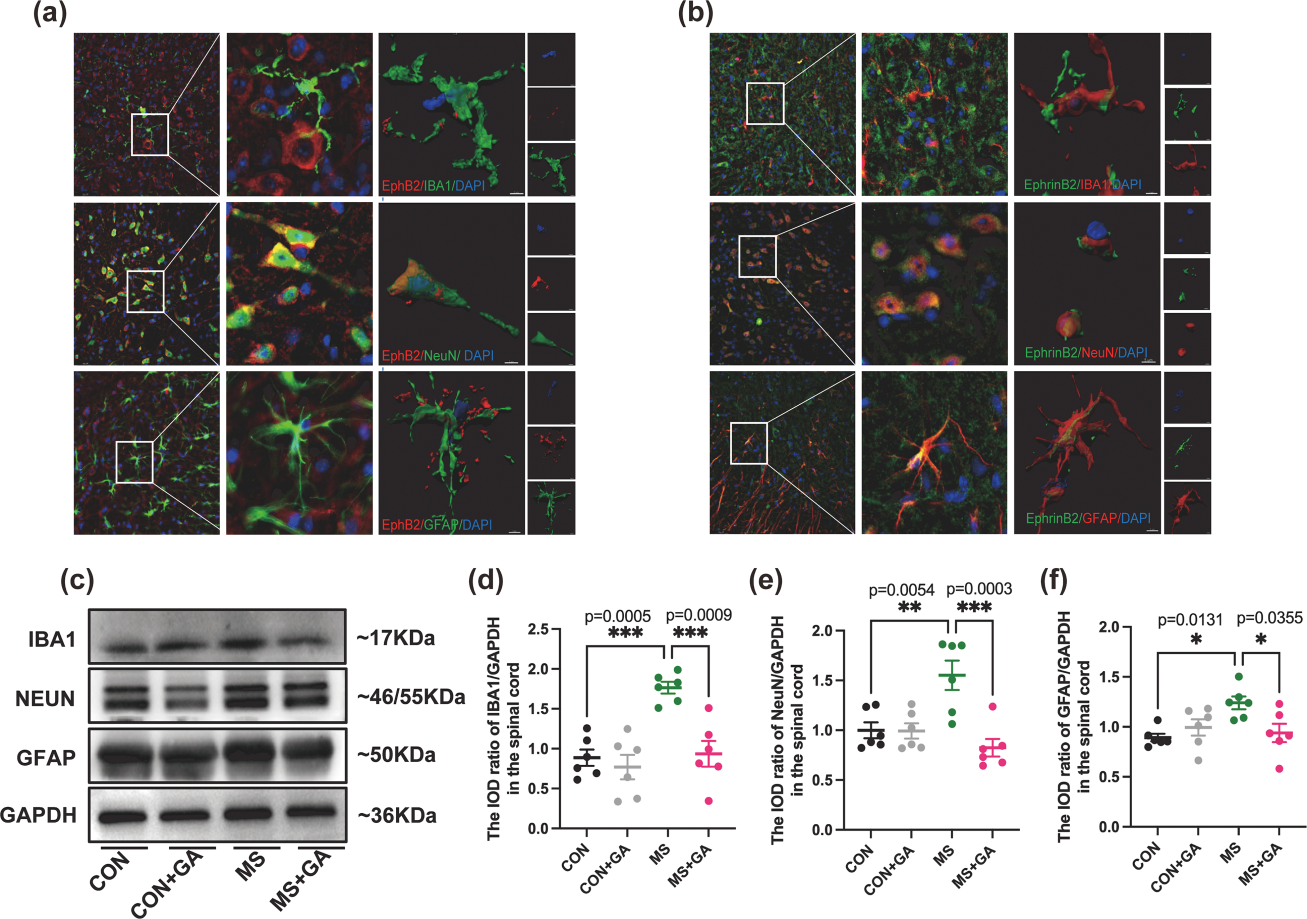
GA reduces the expression of IBA1, NeuN, and GFAP in MS mice. **(a, b)** EphrinB2/EphB2 co-expression with neurons and glial cells in the spinal cord. Representative 3D reconstructions show the co-localization of EphB2 with spinal cord neurons or glial cells **(a)** and EphrinB2 with spinal cord neurons or glial cells **(b)**. NeuN (neuron-specific marker), GFAP (astrocyte-specific marker), and IBA1 (microglia-specific marker) were used for cell type identification.(c-f) Western blot analysis and quantification of the relative gray values of IBA1 **(c)**, NeuN **(e)**, and GFAP **(f)** expression in the spinal cords of control, control+GA, MS, and MS+GA groups. Representative blots **(c)** and quantification of IBA1 **(d)**, NeuN **(e)**, and GFAP **(f)** are shown. Data are presented as mean ± SEM (n=6 mice/group), and statistical significance was determined by one-way ANOVA with Bonferroni’s *post hoc* test (multiple groups). *, *p* < 0.05; **, *p* < 0.01; ***, *p* < 0.001. CON+GA vs. MS: *p* < 0.05. GA: Gallic acid.

To investigate whether EphrinB2/EphB2 activation specifically occurs in neurons and/or glial cells, we conducted a three-dimensional reconstruction analysis to precisely localize EphrinB2/EphB2 expression (**Figures 3a, b**, demonstrating that EphrinB2/EphB2 is expressed in both neurons and glial cells, especially in neurons and astrocytes. What’s more, To further validate these findings, we performed Western blot analysis (**Figure 3c**), which revealed that the spinal cords of MS mice had significantly higher levels of IBA1, NeuN, and GFAP expression than the control group (p < 0.05), indicating that neurons and glial cells were all activated following CRD in MS mice. Conversely, the levels of IBA1, NeuN, and GFAP were considerably lower in the GA-treated MS group than in the MS group (p < 0.05) (**Figures 3d–f**), while no significant changes were observed in these protein levels in the control groups.

### 3.4 Effect of GA on the co-expression of IBA1, NeuN or GFAP with EphrinB2/EphB2 in the spinal cord of mice

**Figure 4:**
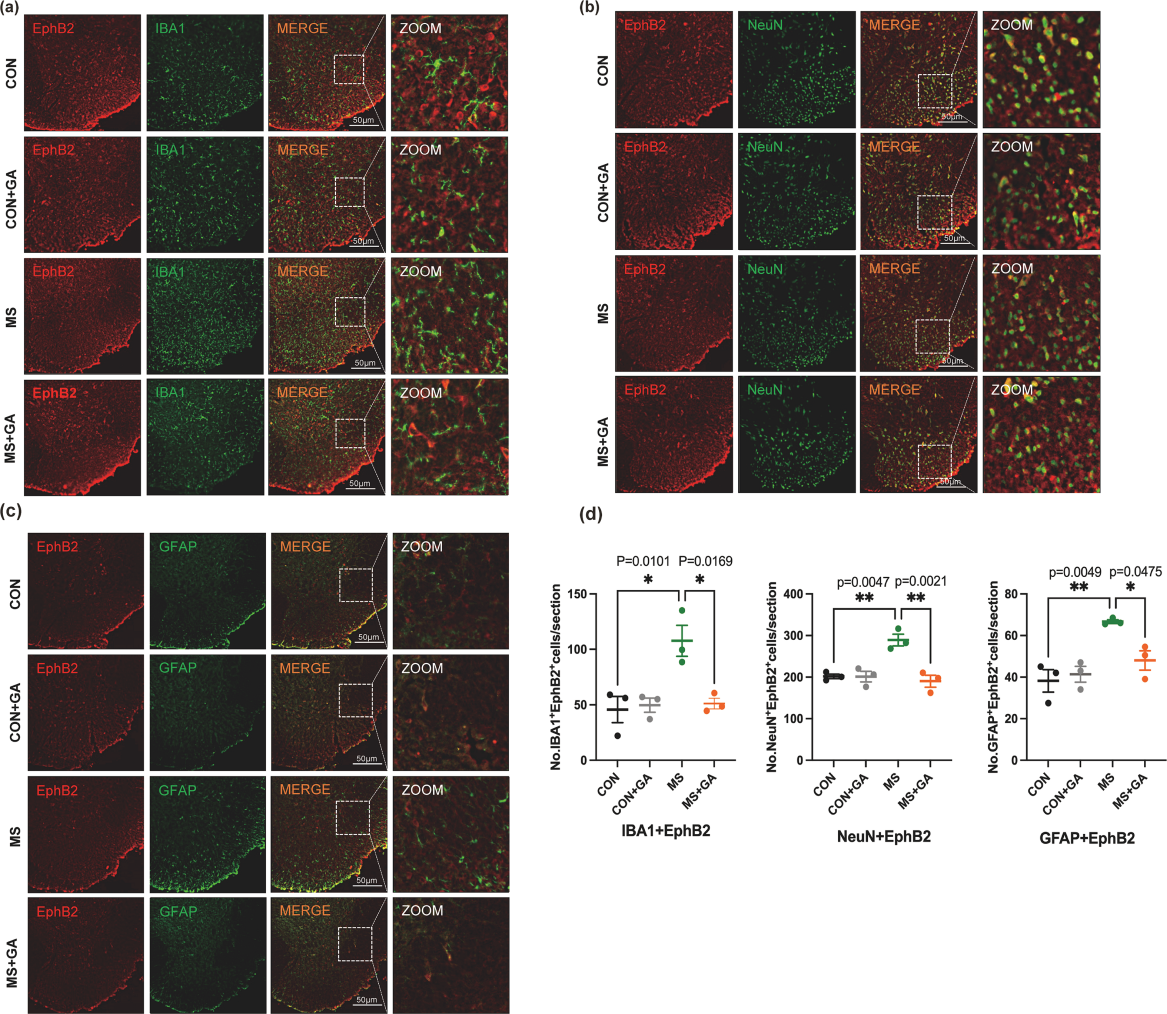
GA decreases the co-expression of IBA1, NeuN or GFAP and EphrinB2/EphB2 in the spinal cord. **(a-d)** Quantitative analysis of double-positive cells for EphB2 co-expressed with IBA1, NeuN, or GFAP in control, control+GA, MS, and MS+GA groups **(d)**. Representative immunofluorescence images depict EphB2 in red and IBA1 **(a)**, NeuN **(b)** or GFAP **(c)** in green. Data are presented as mean ± SEM (n=3 mice/group), and statistical significance was determined using a one-way ANOVA with Bonferroni’s *post hoc* test (multiple groups). *, *p* < 0.05; **, *p* < 0.01. CON+GA vs. MS: *p* < 0.05. Scale bar: 50 μm. GA: Gallic acid.

**Figure 5:**
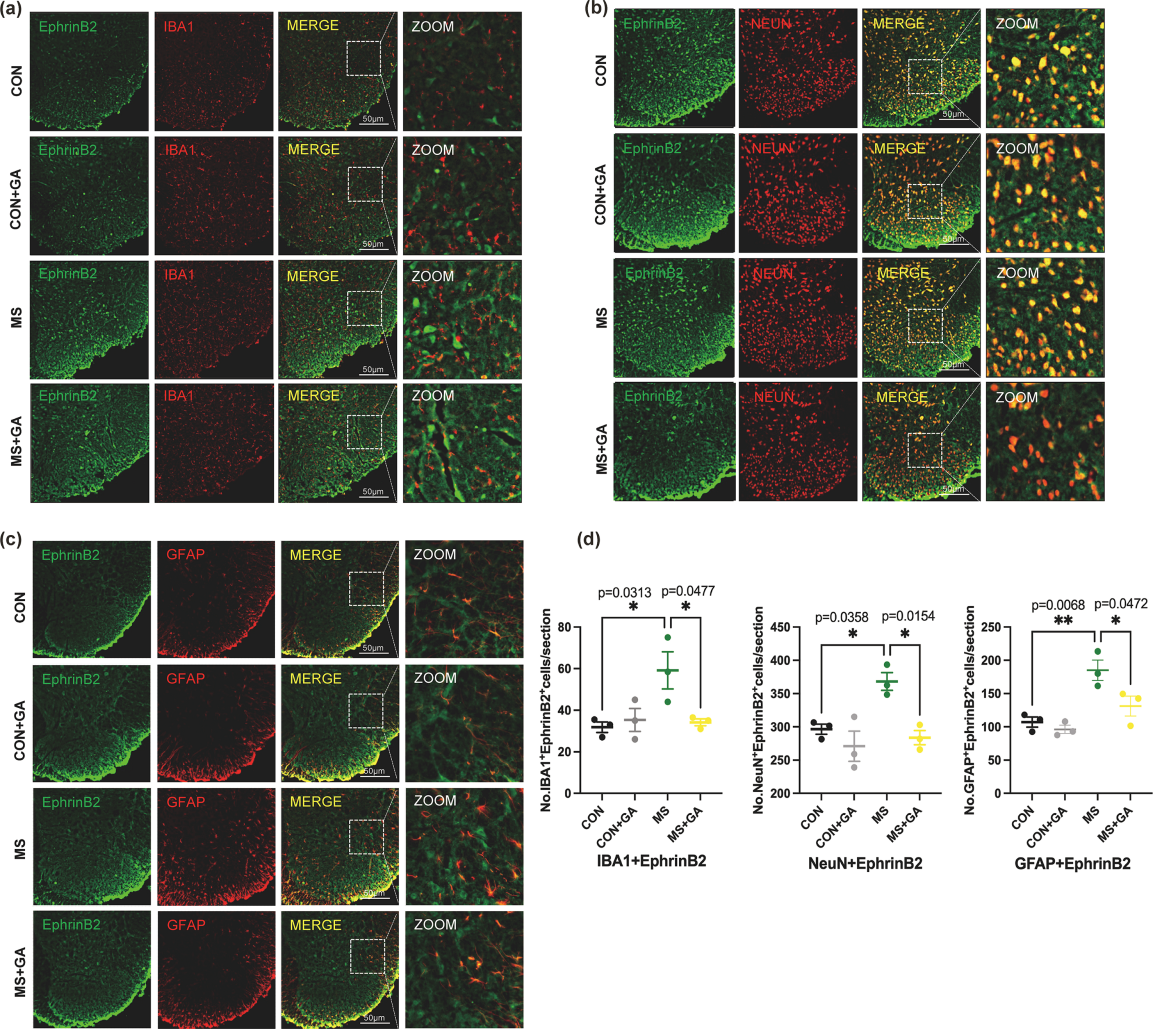
GA decreases the co-expression of IBA1, NeuN or GFAP and EphrinB2/EphB2 in the spinal cord. **(a-d)** Quantitative analysis of double-positive cells for EphrinB2 co-localized with IBA1, NeuN, or GFAP in control, control+GA, MS, and MS+GA groups **(d)**. Representative images depict EphrinB2 in green, with IBA1 **(a)**, NeuN **(b)** or GFAP **(c)** shown in red. Data are presented as mean ± SEM (n=3 mice/group). Statistical significance was determined using a one-way ANOVA and Bonferroni multiple comparisons test. *, *p* < 0.05; **, *p* < 0.01. CON+GA vs. MS: *p* < 0.05. Scale bar: 50 μm. GA: Gallic acid.

Based on the 3D reconstruction localization of EphrinB2/EphB2 with IBA1, NeuN, or GFAP, we quantitatively analyzed the number of double-positive cells for EphrinB2/EphB2 with these markers across each group (**Figures 4a-c****) (****Figures 5a–c**). In the spinal dorsal horn of MS mice, co-expression of EphrinB2 and EphB2 with IBA1, NeuN, or GFAP was considerably higher than in the control group (p<0.05), according to the analysis. In contrast, the MS group treated with GA showed a notable reduction in this co-expression (p < 0.05). There were no discernible changes between the control group and the GA-treated control group (p>0.05) (**Figures 4d****,****5d**). These findings suggest that the expression of neuronal and glial markers (IBA1, NeuN, and GFAP) is closely associated with EphrinB2/EphB2 signaling, and that GA modulates this interaction, influencing signal communication between spinal dorsal horn neurons and glial cells.

### 3.5 GA blocks exogenous EphrinB2-induced EphB2 receptor activation and its resulting visceral hyperalgesia in naive mice

**Figure 6:**
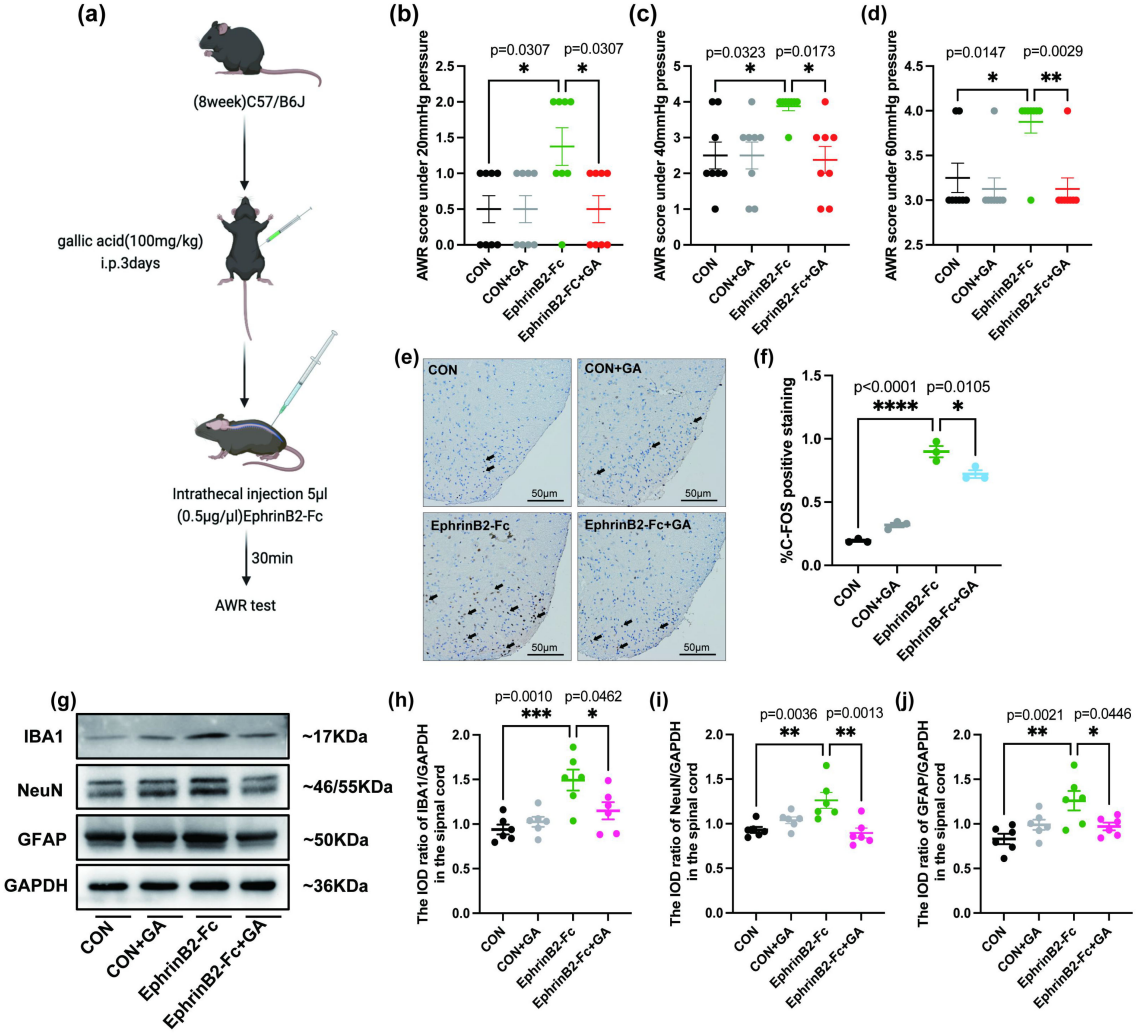
GA alleviates pain behavior and reduces the protein expression in EphrinB2-Fc treated mice. **(a)** Timeline of EphrinB2-Fc modeling and GA treatment. **(b-d)** Effect of GA treatment on pain behaviors in EphrinB2-Fc mice. AWR scores were measured at stimulation pressures of 20 **(b)**, 40 **(c)** and 60 **(d)** mmHg across control, control+GA, EphrinB2-Fc, and EphrinB2-Fc+GA groups. Data are presented as mean ± SEM (n=8 mice/group) and analyzed by One-way ANOVA and Bonferroni multiple comparisons test. *, *p* < 0.05; **, *p* < 0.01. CON+GA vs. MS: *p* < 0.05. **(e, f)** Representative immunohistochemistry images of the spinal dorsal horn showing positive staining **(e)**, along with quantitative analysis of staining intensity **(f)**. Data are presented as mean ± SEM (n=3 or 4 mice/group) and analyzed by One-way ANOVA and Bonferroni multiple comparisons test. *, *p* < 0.05; ****, *p* < 0.0001. CON+GA vs. MS: *p* < 0.0001. **(g-j)** Effect of GA treatment on the expression of IBA1 **(h)**, NeuN **(i)** and GFAP **(j)** in the spinal cord of EphrinB2-Fc mice. Quantitative data and representative images for each marker are shown **(g)**. Data are presented as mean ± SEM (n=6 mice/group) and analyzed by One-way ANOVA and Bonferroni multiple comparisons test. *, *p* < 0.05; **, *p* < 0.01; ***, *p* < 0.001. CON+GA vs. MS: *p* < 0.05. GA: Gallic acid; AWR: Abdominal withdrawal reflex.

Previous studies have shown that EphrinB/EphB binding triggered bidirectional signaling (12, 21). We examined its interaction with EphrinB2/EphB2 through molecular docking (**Figure 2b**), which demonstrated GA’s binding to both the EphrinB2/EphB2 complex and EphrinB2 alone. This finding led us to hypothesize that GA may block exogenous EphrinB2-driven EphB2 activation. To test this, naive C57/B6J mice were given 100 mg/kg of GA for three days in a row. EphrinB2-Fc (0.5 μg/μl, catalog no. 496-EB, R&D Systems, Minnesota, USA) was then injected intrathecally. After that, pertinent behavioral assessments were carried out (**Figure 6a**).

The findings demonstrated that GA-treated mice had significantly lower AWR scores at pressures of 20, 40, and 60 mmHg than the EphrinB2-Fc group (p<0.05) (**Figures 6b–d**). In comparison to the control group, EphrinB2-Fc mice exhibited a considerable increase in C-fos positive staining, which was significantly decreased following GA treatment, according to immunohistochemical analysis (**Figures 6e–f**). Moreover, the expression levels of IBA1, NeuN, and GFAP were notably higher in the EphrinB2-Fc group compared to the controls. In contrast to the EphrinB2-Fc group, these protein levels were considerably lower in the EphrinB2-Fc + GA group (**Figures 6g–j**).

**Figure 7:**
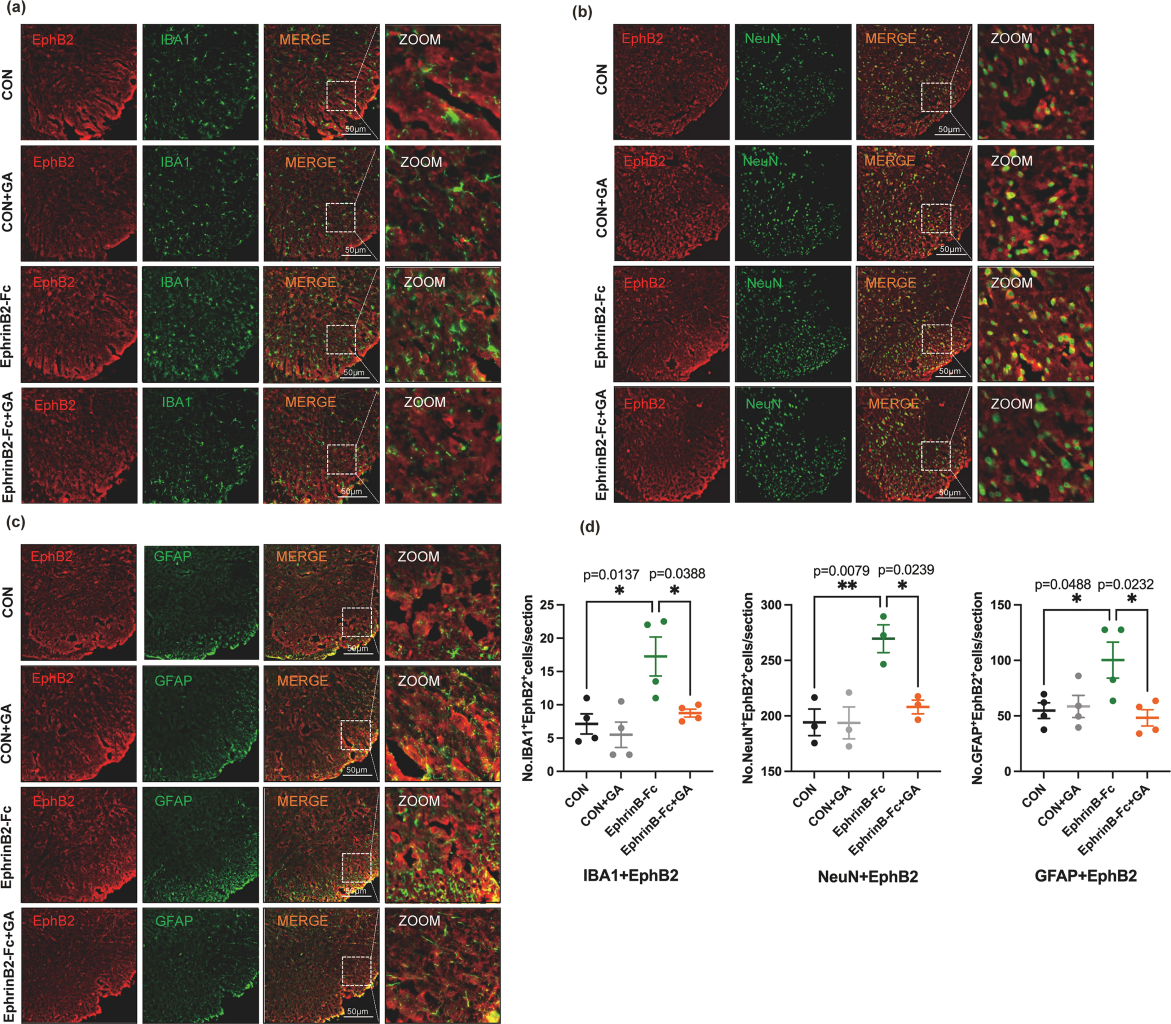
GA blocks exogenous EphrinB2-driven EphB2 activation in naive mice. **(a-d)** Representative immunofluorescence images of the spinal dorsal horn showing co-localization of EphB2 with IBA1 **(a)**, NeuN **(b)** or GFAP **(c)**. Scale bar: 50 μm. **(d)** Quantitative analysis of double-positive cells for EphB2 and IBA1, NeuN, or GFAP in each group. Data are presented as mean ± SEM (n=4 mice/group). Statistical significance was determined using a one-way ANOVA and Bonferroni’s *post hoc* test (multiple groups). *, *p* < 0.05; **, *p* < 0.01. CON+GA vs. MS: *p* < 0.05. GA: Gallic acid.

The co-expression of the EphB2 with IBA1, NeuN, or GFAP was markedly decreased in the EphrinB2-Fc+GA group compared to the EphrinB2-Fc group (**Figures 7a–d**). The collective results implied that GA can block exogenous EphrinB2, reducing the activation of neural and glial markers associated with EphB2 signaling, thereby alleviating visceral pain.

### 3.6 Effect of GA on the expression of NMDA receptors at the spinal cord level in mice

**Figure 8:**
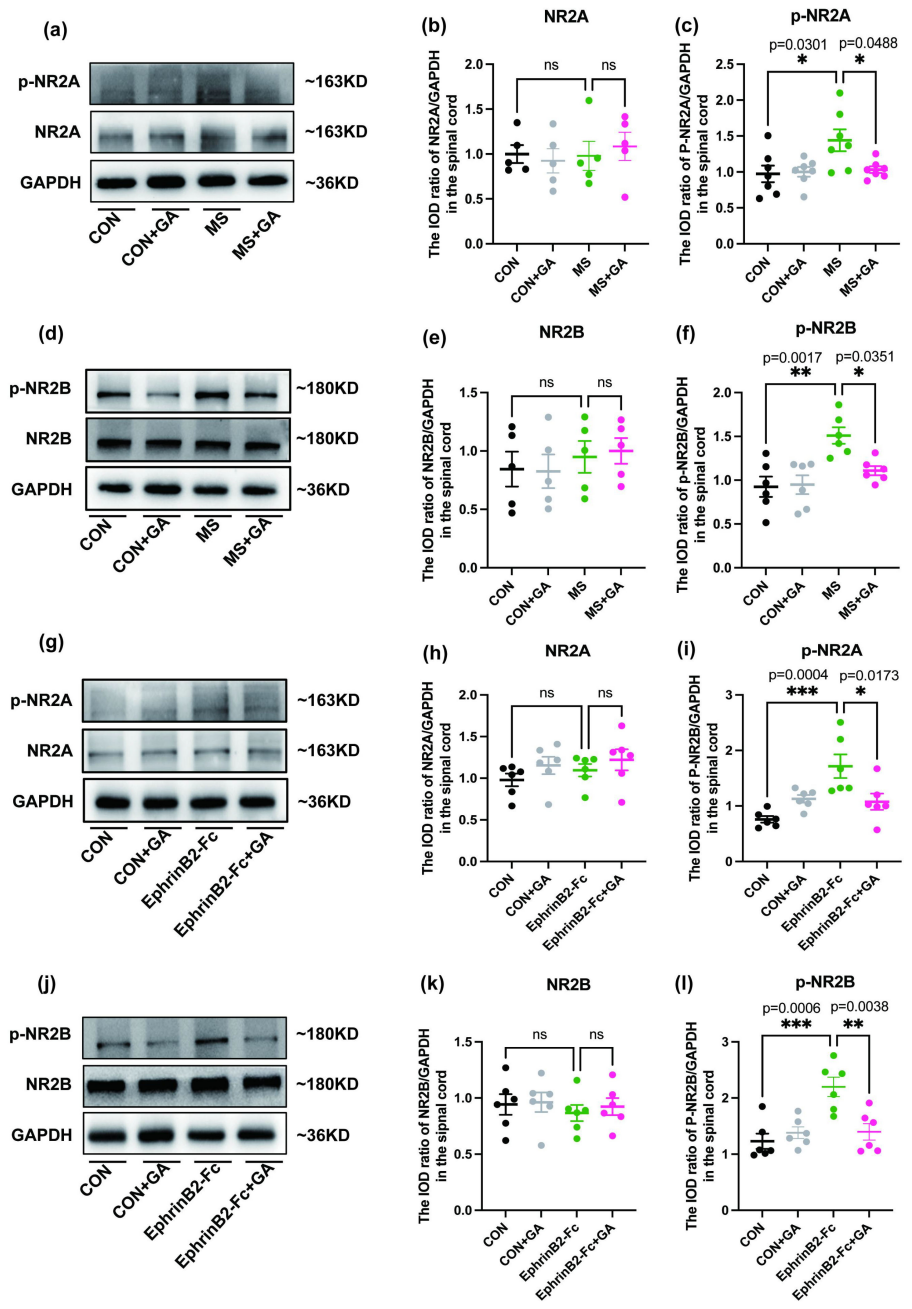
GA affects the expression of NMDAR in the spinal cord of mice. **(a-g)** Western blot analysis of NMDAR expression in the spinal cords of MS mice **(a)** and EphrinB2-Fc mice **(g)** across control, control+GA, MS, and MS+GA groups or control, control+GA, EphrinB2-Fc, and EphrinB2-Fc+GA groups, respectively. (**b, c** and **h, i**) Quantification of the relative gray values of NMDAR expression in the spinal cords of MS mice **(b, c)** and EphrinB2-Fc mice (**h-i**). (**d-f** and **j-l**) Effect of GA treatment on NMDAR expression, presented through representative Western blot images (**d-f** for MS mice, **j-l** for EphrinB2-Fc mice) and their corresponding quantification. Data are expressed as mean ± SEM (n=6 mice/group), and statistical significance was determined using a one-way ANOVA and Bonferroni’s *post hoc* test (multiple groups). *, *p* < 0.05; **, *p* < 0.01; ns: no significance. CON+GA vs. MS: *p*>0.05 **(b,e,h,k)**, *p* < 0.05 **(c,f,i,l)**. GA: Gallic acid; NMDAR:N-methyl-D-aspartate receptor; MS: Maternal separation.

To investigate changes in synaptic plasticity within the spinal cord of MS mice, we examined the expression levels of NR2A, NR2B, phospho-NR2A (p-NR2A), and phospho-NR2B (p-NR2B)(**Figures 8a, d, g, j**). The results showed significantly elevated levels of p-NR2A and p-NR2B in both the MS and EphrinB2-Fc groups compared to the control group (**Figures 8c, f, i, l**), while the expression levels of NR2A and NR2B remained consistent across all groups (**Figures 8b, e, h, k**). These findings suggest that activation of the EphrinB2/EphB2 signaling pathway regulates synaptic plasticity in the mice spinal cord. Furthermore, the expression levels of p-NR2A and p-NR2B in the MS+GA group were significantly reduced than those in the MS group. A similar trend was observed where the pre-injection of GA inhibited the effects of exogenous EphrinB2. All of these works demonstrate that GA inhibition of both endogenous and exogenous EphrinB2/EphB2 signaling pathways could ameliorate abnormal changes in the synaptic plasticity of the spinal cord in mice.

## 4 Discussion

EphrinB2/EphB2 signaling plays a crucial role in various pain models, and its inhibition in the spinal cord is capable of prevent and manage persistent pain (22). Our experimental data suggest the following: (1) MS activates the EphrinB2/EphB2 pathway in the mice spinal cord, increasing neuronal activity, glial cell activation. (2) GA can stably bind to the EphrinB2-EphB2 complex or EphrinB2 receptor, modulating EphrinB2/EphB2 activity and affecting neuronal and glial activation. (3) GA inhibition of EphrinB2/EphB2 signaling pathway decreased the expression of p-NR2A and p-NR2B in MS mice, altered synaptic plasticity, and thus affected signal communication between spinal dorsal horn neurons and glial cells. These findings underscore GA’s potential to modulate EphrinB2/EphB2 signaling and alleviate early stress -induced visceral hyperalgesia.

The EphrinB2/EphB2 signaling pathway is essential for early nervous system development (23) and plays a regulatory role in adult spinal cord injury responses (24). Activation of EphrinB2/EphB2 signaling in dorsal root ganglia and dorsal horn neurons enhances dorsal horn synaptic plasticity and contributes to bone cancer pain (25). In this study, we show that spinal EphrinB2/EphB2 activation is closely linked to visceral hyperalgesia behaviors and increased C-fos expression induced by MS stress. GA, a promising therapeutic candidate, has been shown to inhibit purinergic P2X7 receptors, reducing neuropathic and visceral pain (15, 26). Our molecular docking analysis reveals a novel mechanism by which GA alleviates visceral pain: by blocking EphrinB2/EphB2 signaling, GA significantly reduces visceral hyperalgesia and C-fos expression.

Central sensitization refers to the activity-dependent functional plasticity of spinal neurons and is a primary contributor to behavioral hyperalgesia in pathological states (27). While the nature of visceral hyperalgesia is complex, central sensitization, driven by the heightened excitability of ascending spinal neurons that receive visceral synaptic input, plays a significant role in this phenomenon (28). Recent research indicates that central sensitization involves not only neuronal mechanisms but also glial activation. Microglia and astrocytes are pivotal in detecting pathological events and modulating central nervous system functions (29–31). Nociceptive processes stimulate microglia to release pro-inflammatory cytokines and chemokines, which enhance visceral hyperalgesia by increasing glutamate and reducing gamma-Aminobutyric Acid levels (29, 32), while promoting synaptic transmission by elevating neuronal excitability (29). Activated astrocytes have increased protein kinases, signaling molecules, and cell surface receptors that interact with each other to transmit signals in response to pain (32). In our study, Western blotting revealed upregulation of IBA1, NeuN, and GFAP in MS mice following CRD, aligning with previous studies on spinal glial activation during pain (29, 33). Moreover, previous studies have demonstrated that ELS can lead to diverse and stage-dependent alterations in neuronal and glial activation throughout the central nervous system, including modifications in neuronal subtypes as well as glial morphology and reactivity (34–37). The influence of MS on central nervous system development is likely a complex and dynamic process. It is possible that MS predisposes the central nervous system to aberrant neuronal and glial activation upon exposure to stimuli in adulthood. This mechanism may account for the phenomena observed in our experiments. In the present study, we found that mice subjected to MS displayed enhanced activation of both neurons and glial cells following CRD when compared with normally reared mice. However, it should be noted that we did not evaluate potential baseline differences in the numbers of neuronal and glial cells between MS and control mice in the absence of CRD stimulation. Immunofluorescence 3D reconstruction also showed substantial EphrinB2/EphB2 expression in astrocytes and neurons, with lower expression in microglia (**Figures 3e, f**). GA treatment significantly reduced EphrinB2/EphB2 co-expression with IBA1, NeuN, and GFAP in the spinal dorsal horn, suggesting that GA may alleviate visceral hyperalgesia by inhibiting the EphrinB2/EphB2 pathway mainly modulating its astrocyte and neuron activity.

EphrinB2/EphB2 may interact with NMDARs to mediate synaptic enhancement (8, 38). For instance, functional synaptic plasticity is enhanced in IBS rat models and can be blocked by ifenprodil, a selective NR2B antagonist. NR2B phosphorylation is promoted by EphrinB2-Fc in IBS rats, which may facilitate prolonged neuronal activation by upregulating the expression of immediate early genes linked to synaptic plasticity, such as C-fos (8). It is generally accepted that the most highly expressed NMDARs regulatory subunits in the central nervous system are the NR2A and NR2B subunits (39). However, more research has focused on NR2B (mainly enriched at extrasynaptic sites) than on NR2A (predominantly synaptic) in the context of neurological diseases (40, 41). In early development, NR2B-containing NMDARs are dominant, whereas NR2A-containing NMDARs gradually increase in expression, eventually surpassing NR2B in adulthood (41–43). Additionally, NR2A has a higher affinity for glutamate than NR2B, meaning that synaptic glutamate release in adult neurons preferentially stimulates NR2A-containing NMDARs (41). To further examine GA’s role in alleviating visceral hyperalgesia induced by MS stress, we evaluated the phosphorylation levels of NR2A and NR2B in the spinal cords of mice subjected to MS and those activated by exogenous EphrinB2 via Western blotting. Our results showed a reduction in p-NR2A and p-NR2B expression in the spinal cords of mice following GA treatment. These results provide credence to the theory that GA affects synaptic function in the dorsal horn of the spinal cord in mice by blocking the EphrinB2/EphB2 pathway.

Based on our findings and prior studies, we propose that when the intestine experiences noxious stimulation, such as CRD, primary sensory terminals in the intestine transmit injury signals to neurons in the spinal dorsal horn. This signaling is accompanied by an upregulation of EphrinB2 in presynaptic neurons, subsequently activating EphB2 receptors on postsynaptic neurons in MS mice. Additionally, activated EphrinB2 may influence NMDARs function. CRD also induces the upregulation of EphrinB2 ligands and EphB2 receptors in astrocytes and microglia, facilitating interactions among astrocytes, microglia, and both presynaptic and postsynaptic neurons via the EphrinB2/EphB2 signaling pathway. This interaction establishes a positive feedback loop, amplifying pain signals within the neural network (Hypothesis illustration) (44). Furthermore, GA may inhibit EphrinB2 activity, leading to a reduction in EphB2 expression in both neurons and glial cells. This decrease in EphB2 levels could modify synaptic plasticity in astrocytes and microglia, ultimately impacting pain signaling pathways.

This study has several limitations that warrant consideration. First, our focus was primarily on the spinal cord, which may overlook the potential effects of GA on other regions such as the intestine, brain, and dorsal root ganglia, thereby limiting our understanding of GA’s broader physiological impacts. Second, while microglial responses are generally early and transient in the development and maintenance of pain, astrocyte activation occurs later and persists for a longer duration (45–47). However, the CRD test employed in our experiments only assessed short-term pain responses in mice, leaving the long-term mechanisms of glial cell and neuronal involvement in chronic visceral pain unclear. Third, AWR scoring system provides a valuable and direct behavioral correlate of visceral nociception. The AWR is inherently subjective and can be influenced by observer bias, despite rigorous standardization of the scoring protocol. The electrophysiological recording of abdominal muscle activity via electromyography (EMG) is considered the gold standard for quantifying visceral hypersensitivity. EMG provides a direct, objective, and quantifiable measure of the neural efferent output in response to noxious colorectal distension, offering superior sensitivity and a continuous data scale. Future investigations would benefit from correlating behavioral AWR scores with simultaneous EMG recordings to provide a more comprehensive and objective assessment of the nociceptive response. Fourth, this study did not account for sexual dimorphism in visceral hyperalgesia. Consequently, subsequent studies are crucial to specifically examine sex-specific differences in pain-induced reactivity, particularly in female mice. Finally, although GA has a broad therapeutic window with few toxicity at low doses, mild toxicity may occur at higher doses (11, 48). Our use of a single dose of 100 mg/kg may not fully capture the dose-dependent therapeutic and toxic effects. Additionally, GA’s low bioavailability and rapid elimination present challenges to maintaining effective therapeutic levels, which may limit its clinical application (48). Future research should focus on developing GA nanoparticles and targeted delivery systems to improve its bioavailability and efficacy in clinical contexts.

## 5 Conclusion

Despite the limitations outlined, our studies demonstrates that GA effectively modulates the EphrinB2/EphB2 signaling pathway, thereby regulating visceral hyperalgesia in MS mice. These findings provide a foundational basis for further investigation into potential therapeutic strategies for managing visceral hyperalgesia.

## Data Availability

The original contributions presented in the study are included in the article/**Supplementary Material**. Further inquiries can be directed to the corresponding author.
