# Supplementary material for: Gallic acid alleviates visceral hyperalgesia following maternal separation in mice by inhibiting EphrinB2/EphB2 signaling mediated activation of neurons and glial cells
Source: Front Immunol. 2025 Nov 27;16:1698744. doi: 10.3389/fimmu.2025.1698744 (PMC12695550; doi:10.3389/fimmu.2025.1698744)

Supplementary Figure 2(c).

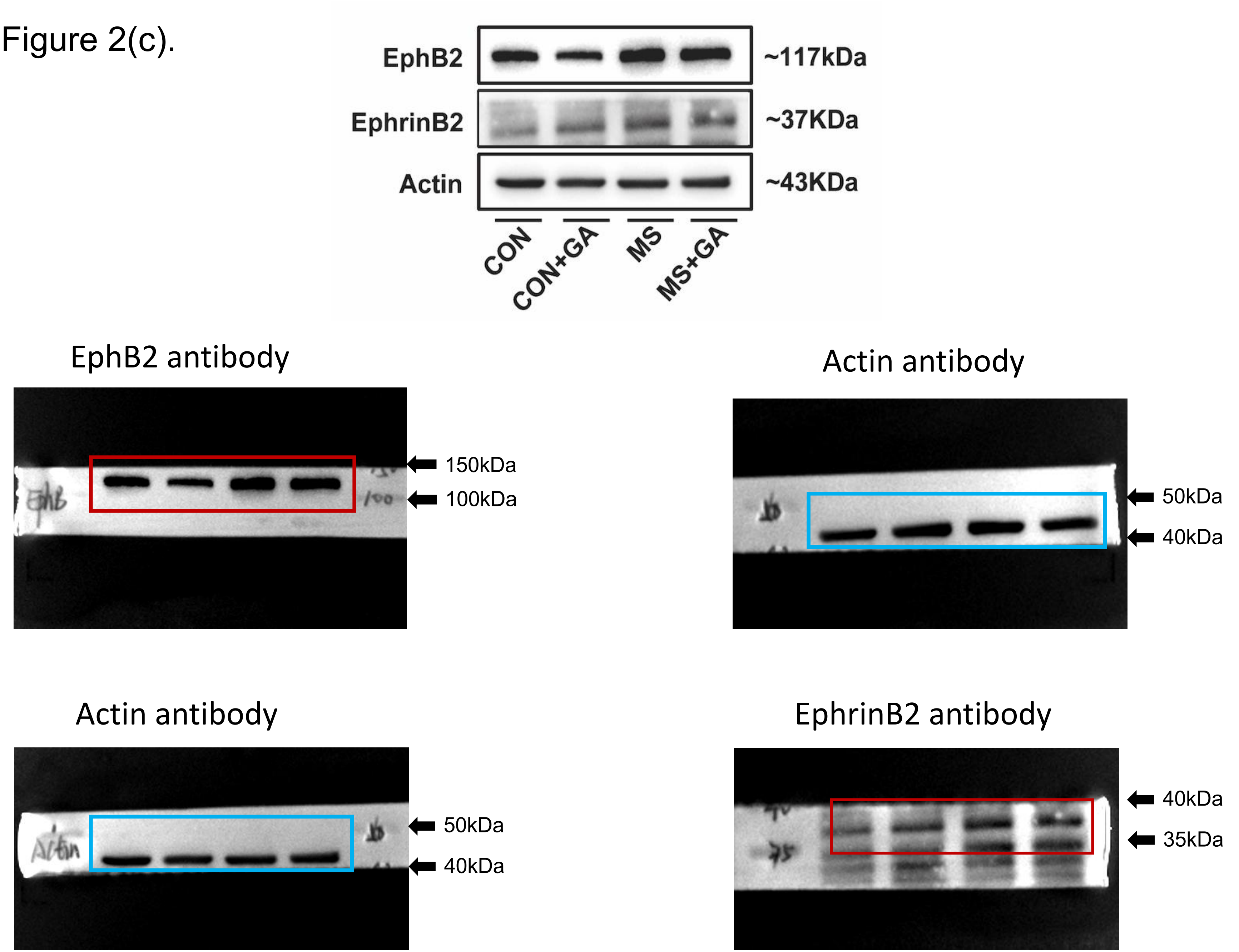

Supplementary Figure 3(c).

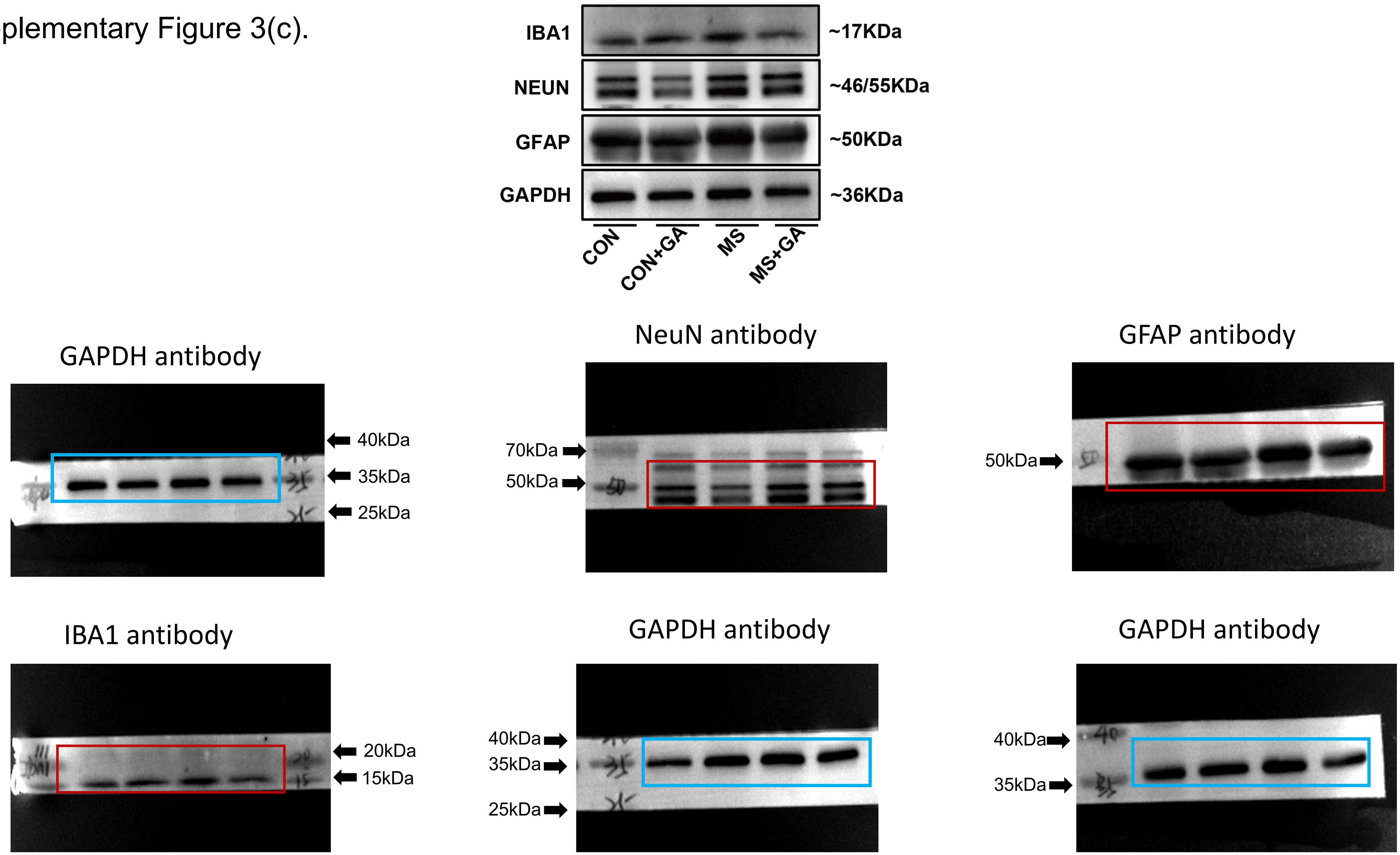

Supplementary Figure 6(g).

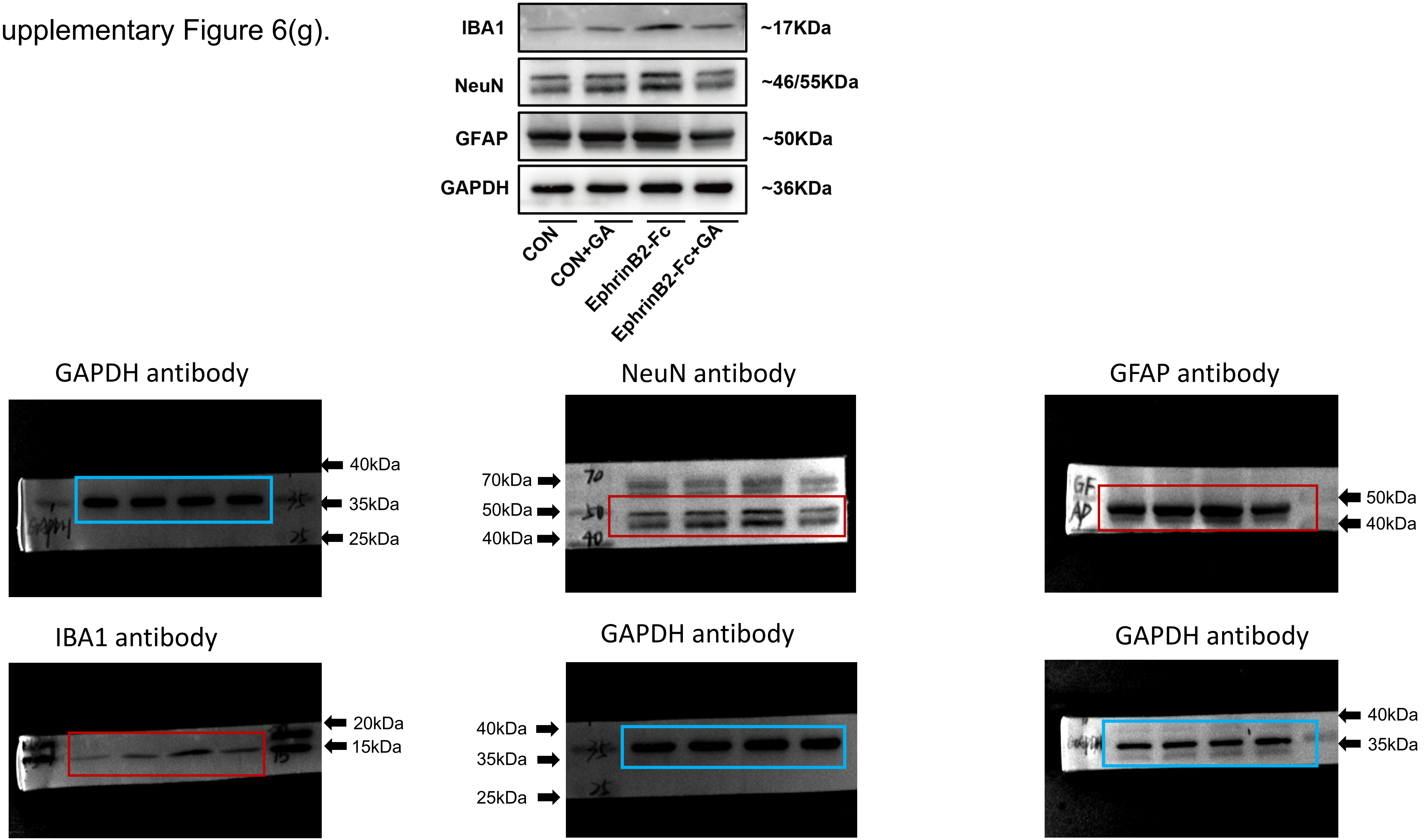

Supplementary Figure 8(a).

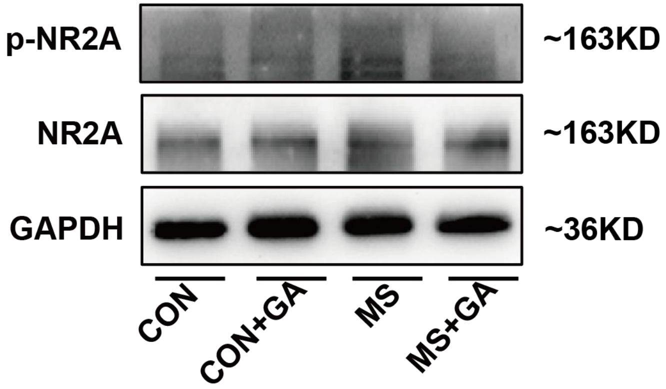

p-NR2A antibody

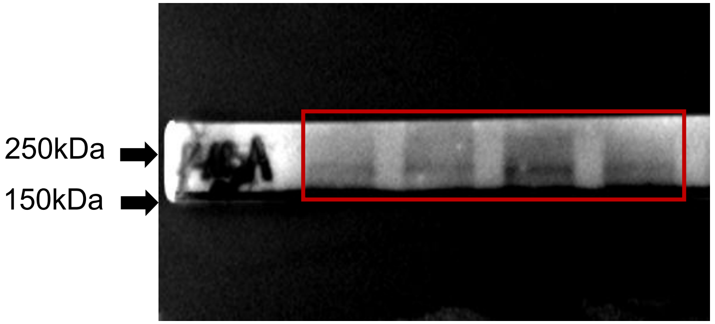

NR2A antibody

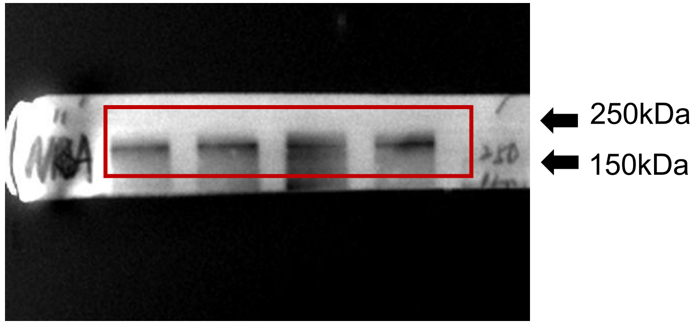

GAPDH antibody

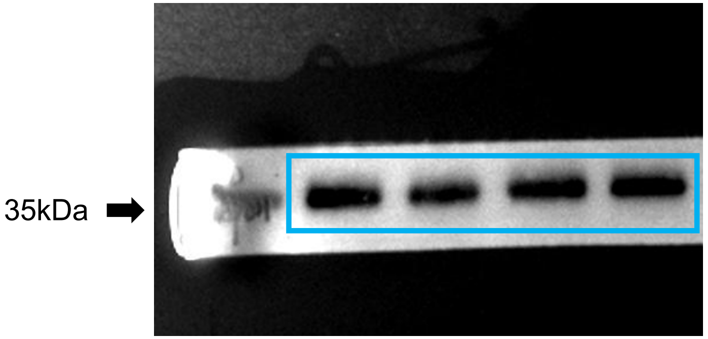

GAPDH antibody

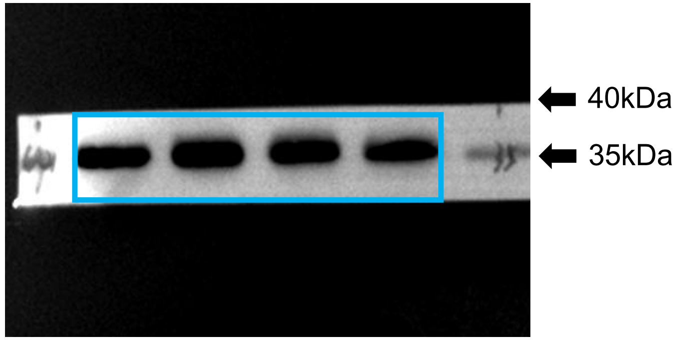

Supplementary Figure 8(d)

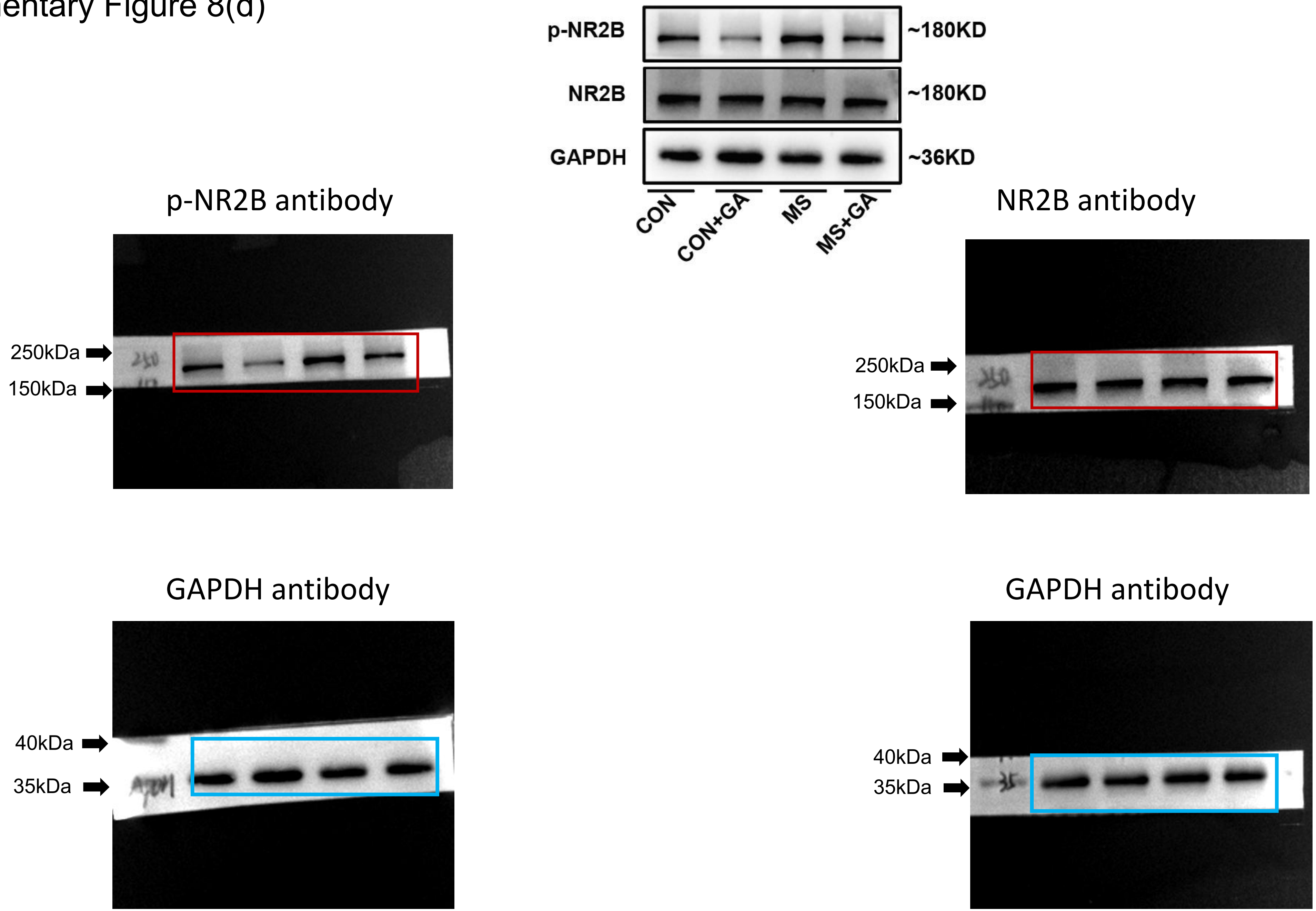

Supplementary Figure 8(g).

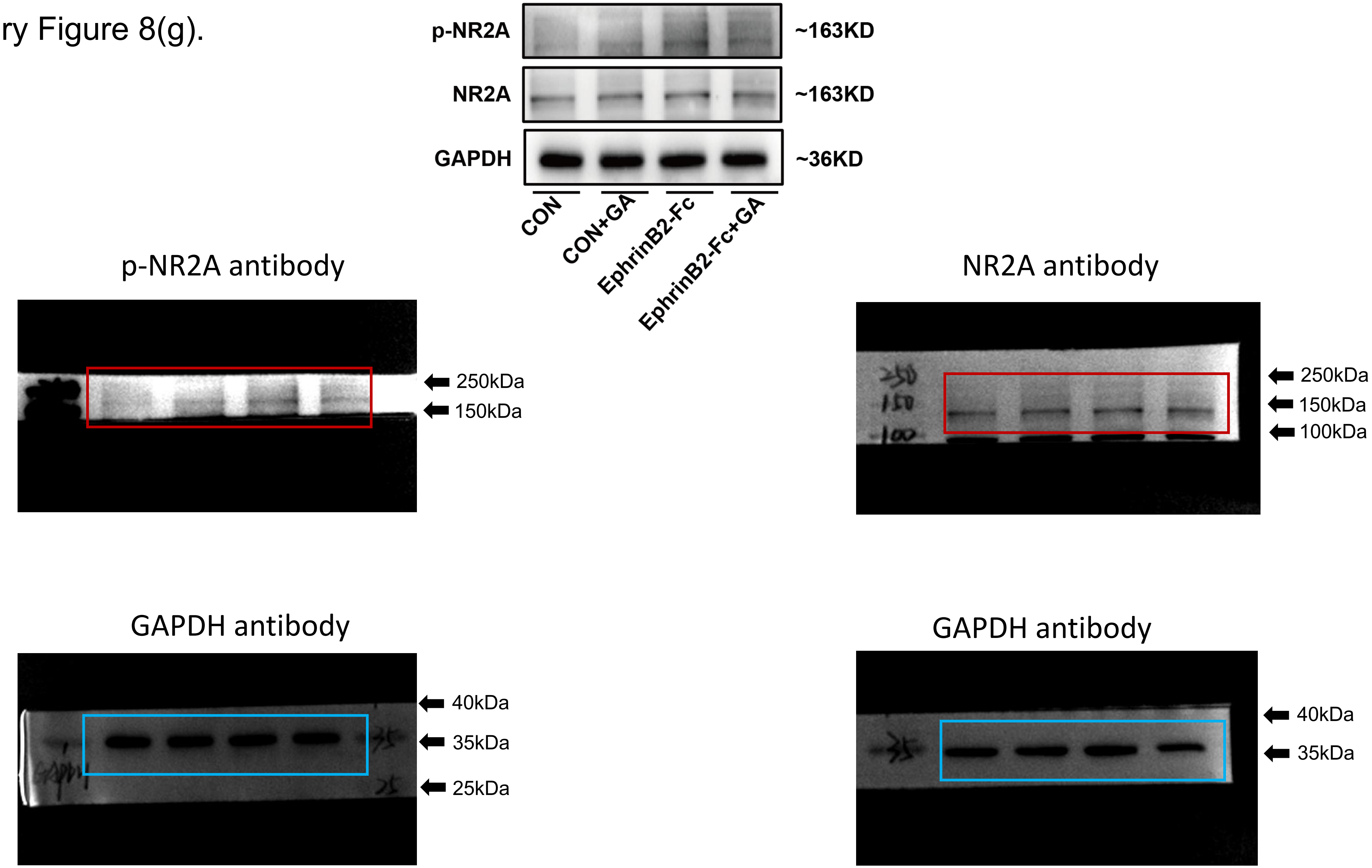

Supplementary Figure 8(j).

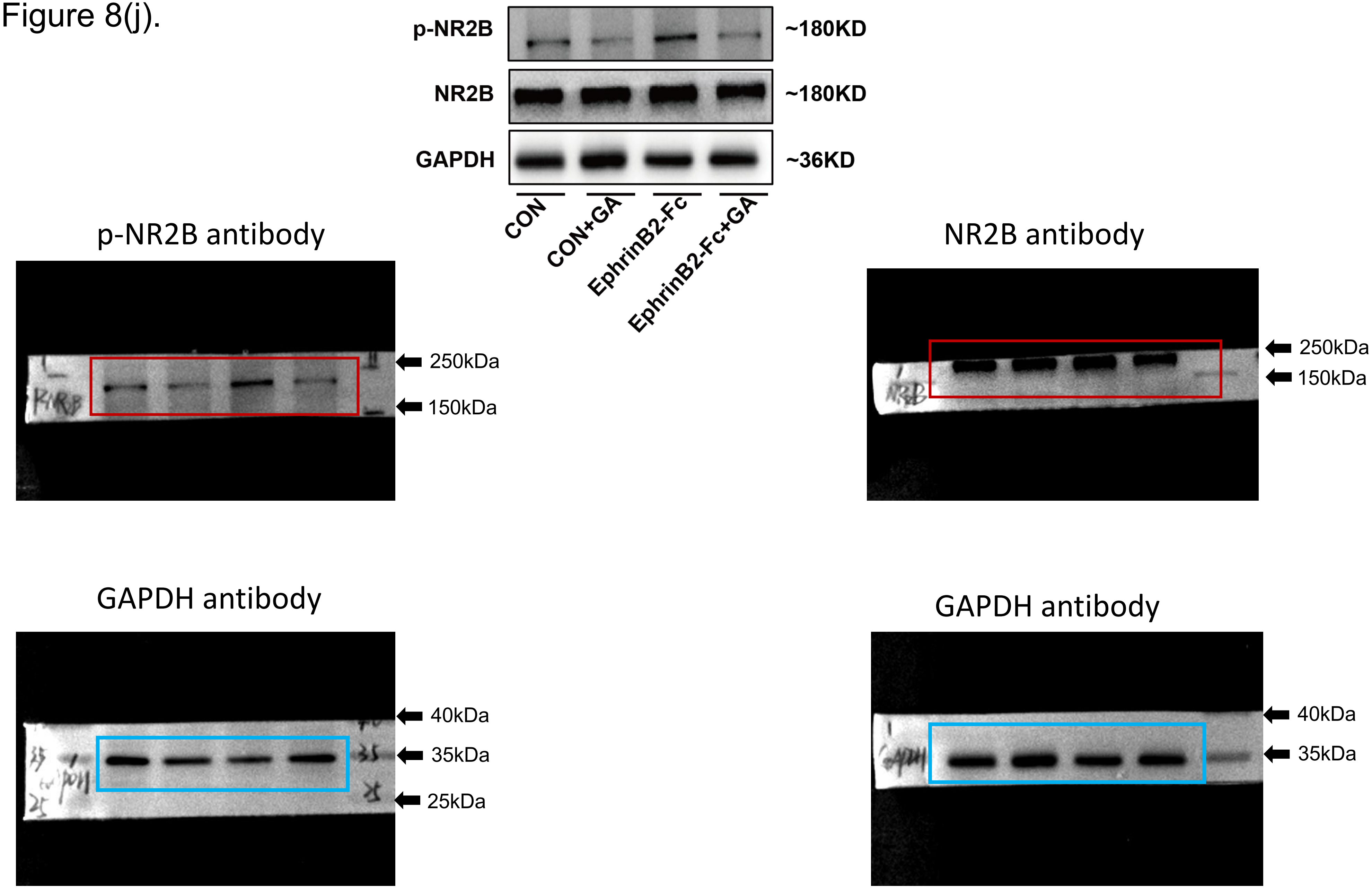

Supplement: Supplementary file 1 [file SupplementaryFile1.pdf]
